# Impaired empathy and increased anger following social exclusion in non-intoxicated opioid users

**DOI:** 10.1007/s00213-019-05378-x

**Published:** 2019-11-05

**Authors:** Molly Carlyle, Megan Rowley, Tobias Stevens, Anke Karl, Celia J. A. Morgan

**Affiliations:** Psychopharmacology and Addiction Research Centre (PARC), University of Exeter, Washington Singer Building, Perry Road, Exeter, EX4 4QG UK

**Keywords:** Opioids, Addiction, Empathy, Social pain, Social cognition, Cortisol

## Abstract

**Rationale:**

Social functioning is modulated by the endogenous opioid system. In opioid use disorder, social functioning appears disrupted, but little research has delineated the nature of these deficits and their relationship to acute opioid use.

**Objectives:**

The current study aimed to assess both emotional and cognitive empathy, along with subjective and physiological responses to social exclusion in opioid users who were either acutely intoxicated or non-intoxicated from using opioids.

**Methods:**

Individuals on an opioid substitution medication (OSM) were divided into ‘intoxicated users’ (had taken their OSM the same day as testing, *n* = 20) and ‘non-intoxicated users’ (had taken their OSM > 12 h ago, *n* = 20) and compared with opioid-naïve controls (*n* = 24). Empathy was assessed using the multifaceted empathy test and self-report questionnaire. Participants also underwent a period of social exclusion (Cyberball Game) and completed measures of mood and physiological responses (salivary cortisol and heart rate).

**Results:**

Non-intoxicated users had significantly lower emotional empathy (the ability to experience others’ emotions), as well as greater anger after social exclusion when compared with the intoxicated users and controls. Anger did not change with social exclusion in the intoxicated user group and cortisol levels were lower overall.

**Conclusions:**

Reduced ability to spontaneously share the emotions of others was reported in non-intoxicated users, particularly regarding positive emotions. There was some support for the idea of hyperalgesia to social pain, but this was restricted to an enhanced anger response in non-intoxicated users. Equivalent rates of empathy between the intoxicated users and controls could indicate some remediating effects of acute opioids.

**Electronic supplementary material:**

The online version of this article (10.1007/s00213-019-05378-x) contains supplementary material, which is available to authorized users.

## Introduction

The misuse of opioids is a growing global concern, with approximately 34 million users worldwide and recent reports of a dramatic increase in overdose rates (United Nations of Office on Drugs and Crime 2018). As well as high rates of mortality, opioid misuse has other health-related consequences, such as increased rates of HIV, hepatitis C and neonatal abstinence syndrome (National Institute on Drug Abuse 2018). Understanding the factors that initiate and maintain opioid use disorder is thus imperative from a public health perspective. Much work has focused on the biological and behavioural mechanisms of opioid addiction; however, research into the role of psychosocial factors is comparatively sparse (Heilig et al. 2016). It is well understood that social factors including social deprivation and interpersonal trauma can predict and maintain addiction (Gerra et al. 2014; Heffernan et al. 2000; Kendler et al. 2014; Lake et al. 2015; MacGregor and Thickett 2011; Naqavi et al. 2011). Opioids may be used in part to compensate for difficulties in emotion regulation (Moustafa et al. 2018; Wolff et al. 2016). Additionally, high rates of social marginalisation, ostracism and discrimination towards addicted individuals (Barry et al. 2014) may perpetuate deficits in social functioning and could contribute to the maintenance of opioid use.

Neurobiologically, the endogenous opioid system plays a role in social functioning (see Machin and Dunbar 2011, for a review) and is involved in empathy (Rutgen et al. 2015), which has a uniquely social purpose (Panksepp and Panksepp 2013; Pearce et al. 2017). Empathy is crucial for interpersonal relationships and bonding: impairments in the ability to empathise are observed in disorders such as autism spectrum disorder (Baron-Cohen and Wheelwright 2004) and schizophrenia (Green et al. 2015), and are related to difficulties in social functioning (Baron-Cohen and Wheelwright 2004). Impaired empathy in people with substance use disorders has also been reported (Ferrari et al. 2014). Two pivotal aspects of empathy are ‘emotional empathy’, referring to the ability to vicariously feel the emotional state of others, and ‘cognitive empathy’, which refers to the ability to identify and understand the emotional state of others (sometimes referred to as ‘theory of mind’) (Baron-Cohen and Wheelwright 2004; Blair 2005). Impairments in emotional empathy have been observed in drug users generally (Ferrari et al. 2014), alcohol users (Maurage et al. 2011) and stimulant users (Kroll et al. 2018a, 2018b; Preller et al. 2014). Two studies with chronic opioid users have similarly reported impairments in emotional empathy using a subjective questionnaire among methadone- and diacetylmorphine-maintained individuals (Stange et al. 2017; Tomei et al. 2017) but a further study failed to replicate these findings (Kroll et al. 2018a). The ability to empathise can be affected by situational factors including psychosocial stress, affective state, and socioeconomic status (Kanske et al. 2017), where acute opioid intoxication state may also be important to understand impairments in empathy within the context of wider social stress.

Opioid drugs may also affect social functioning by altering responses to difficult social events. Acutely, exogenous opioids have shown to alleviate the experience of both physical and emotional pain (Bershad et al. 2016; Inturrisi 2002; Stein et al. 2007). The latter is termed ‘emotional analgesia’ and is thought to be a protective mechanism from emotional pain, and is associated with reductions in subjective distress and cortisol following social exclusion (Bass et al. 2014). ‘Social’ pain is used to refer to a specific form of emotional pain, such as the painful feelings following an unpleasant social event like bullying, social rejection or exclusion (Eisenberger 2015). Both social and physical pain have overlapping neural mechanisms (however see Iannetti et al. 2013, for a review of the differences). Similar to physical pain, the brain responds to social pain (exclusion) by releasing endorphins to buffer against the unpleasant emotional experience (Hsu et al. 2013).

Pain perception is altered following chronic use of opioid drugs. Studies have consistently reported a heightened sensitivity to physical pain in chronic opioid users (Compton et al. 2001; Higgins et al. 2018; Mao 2002; Marion Lee et al. 2011; Pud et al. 2006). Increased opioid tolerance via the downregulation of endogenous opioid receptors has been suggested to underpin opioid-induced hyperalgesia (Higgins et al. 2018; Mao 2002). As physical and social pain share some similar neural mechanisms (Eisenberger 2015; Hsu et al. 2013), it is plausible to suggest that alterations in opioid receptor function could similarly cause a heightened sensitivity to social, as well as physical, pain. To our knowledge, only one study has investigated the link between chronic opioid use and the experience of social pain in non-intoxicated opioid users, and found a heightened cortisol response to social exclusion (Kroll et al. 2019). We do not yet know how being acutely intoxicated affects response to social exclusion and empathy in opioid users, which may be a powerful factor in maintaining problematic substance use.

Therefore, the current study aimed to investigate alterations in social functioning by measuring empathy and responses to social exclusion among individuals with histories of chronic opioid use. We aimed to examine both the acute and long-term effects of opioids in people prescribed an opioid substitution medication (OSM), by testing people intoxicated with OSM at the time of testing, and people who had not taken their medication for at least 12 hours. Based on previous research showing deficits in empathy in opioid users, we hypothesised that both of the opioid user groups would show impairments in empathy; however, given evidence that opioid intoxication is associated with impaired emotional empathy (Stange et al. 2017; Tomei et al. 2017), we predicted that emotional empathy would be most impaired in the intoxicated group. Secondly, we hypothesised that the intoxicated user group would have a dampened response to social exclusion—based on the analgesic effects of opioids and the assertion that physical and social pain are related. We further predicted that the non-intoxicated user group would be more subjectively affected by social exclusion given the hyperalgesia to physical pain seen in non-intoxicated opioid users.

## Method

### Design and participants

Sixty-four participants (39 males; 24 females; 1 non-binary) aged 22–67 (*M* = 42.69, *SD* = 11.54) were recruited into the study. Forty were opioid users currently stabilised on OSM (methadone or buprenorphine), and all had histories of illicit heroin use. Of these, 20 individuals took their opioid prescription in the morning of the study (intoxicated group), and 20 individuals had taken their prescription > 12 h ago (non-intoxicated group). Group membership was validated with tests of salivary opioid levels. The remaining 24 individuals were opioid-naïve controls with no history of opioid use. Groups were matched in age, gender and verbal IQ. Participants were recruited via word of mouth and advertisements in drug services and employment/training agencies.

The study was a mixed design. Inclusion criteria for the opioid groups were a prolonged history of opioid use and currently taking daily OSM. General inclusion criteria were being a minimum of 18 years old and a fluent English speaker. Exclusion criteria were neurological conditions, history of severe mental health issues, diagnosis of a physical illness that directly influences cortisol activity (i.e. Cushing or Addison disease), taking oral steroid medication and pregnancy. Individuals were excluded from the control group if they had any history of opioid use. Participants were asked to abstain from alcohol and drugs 24 h prior to their study session and abstain from smoking or eating for 45 min prior to their session. The study was reviewed by the institutional ethics committee and was conducted in accordance with the Declaration of Helsinki, all participants gave written, witnessed, informed consent.

### Measures

#### Multifaceted empathy test (MET, see Fig 1) (Dziobek et al. 2008)

This computerised task indexes cognitive and emotional empathy. Forty photographs of people with emotionally charged expressions are given in eight blocks consisting of ten pictures each. In half of these blocks, participants are asked to identify the correct emotion of the subject in each scene (cognitive empathy). In the other half, participants were asked to rate how much they empathise with the individual in each scene (emotional empathy). Each image was presented until the participant gave a response, and participants were asked to respond as quickly as possible. The task lasted approximately 15 min. Responses for cognitive empathy were the total count of correctly identified emotions, while responses for emotional empathy were the mean empathy score.

**Fig. 1:**
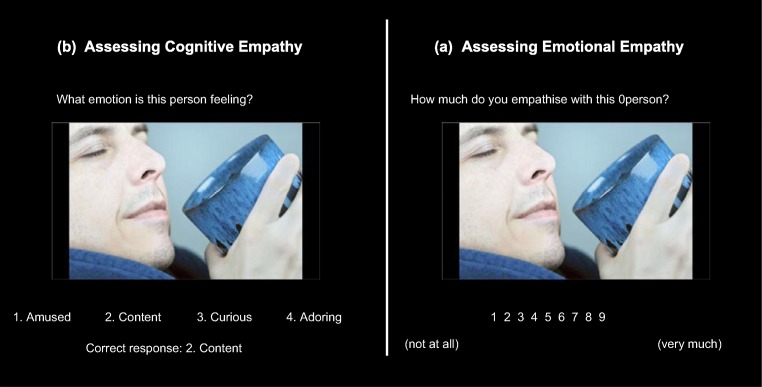
Differential blocks assessing cognitive and emotional empathy in the MET. **a** For cognitive empathy, participants were required to pick one of four emotion labels. **b** For emotional empathy, participants were asked to rate how much they empathised (which they were instructed means ‘feel what they are feeling’) with the subject in the photo using a 9-point Likert Scale (1 = not at all; 9 = very much). Image taken with permission from the task creator (Dziobek et al. 2008)

#### The Cyberball Game (Williams et al. 2012)

This is a computerised ball-tossing game shown to simulate social exclusion. Participants are told that they are playing real people on a virtual network in a mental visualisation experiment, yet unbeknown to them the other players are fictitious and were set up to socially exclude them. In the present study, the Cyberball Game contained four players and had two conditions that simulated either social inclusion or exclusion. There were two games: inclusion followed by exclusion, and each game lasted between 2 and 4 min. Each condition had approximately 60 ball throws between the four players. In the social inclusion condition, participants were over-included and received 20 ± 1 (~ 33.3%) of 60 ball throws. In the exclusion game, participants received exactly 6 ± 1 (~ 10%) of 60 ball throws.

Affective and physiological responses to social inclusion and exclusion were recorded after each game with the Post-ostracism Cyberball Questionnaire (Williams et al. 2002), which assessed mood and basic psychological needs (see supplementary material (SM1) for more details)

#### Physiological measures

Seven saliva samples were collected by passive drool method. Participants were required to provide approximately 2 ml of saliva, which was immediately stored at − 80 °C until analysis using enzyme-linked immunosorbent assay (ELISA) kits to assess cortisol levels, as well as levels of methadone, buprenorphine and opiates (baseline sample only). Heart rate was also assessed alongside each saliva sample (see SM1 for details).

### Questionnaires

Trait empathy was assessed using the Interpersonal Reactivity Index (IRI; Davis 1980), which consists of four different subscales: two characterise emotional empathy (empathic concern; personal distress), and two characterise cognitive empathy (perspective taking; fantasy scale). The Life Events Checklist Version 5 (LEC-5; Weathers et al. 2013) was used to assess trauma by measuring past exposure to any stressful or traumatic life events, and how proximal these events were to the participant (adapted to include age). Loneliness was measured using the UCLA Loneliness Scale (Russell 1996) which assesses feelings of social isolation and loneliness. Craving was assessed using three single items of drug liking, wanting and motivation to obtain opioid drugs (Pool et al. 2016). Verbal IQ was assessed using the Spot-the-Word Test (Baddeley et al. 1993). Mood at baseline was also assessed using the Positive and Negative Affect Schedule (Watson et al. 1988). See SM1 for further details of each psychometric measure.

### Procedure

**Fig. 2:**
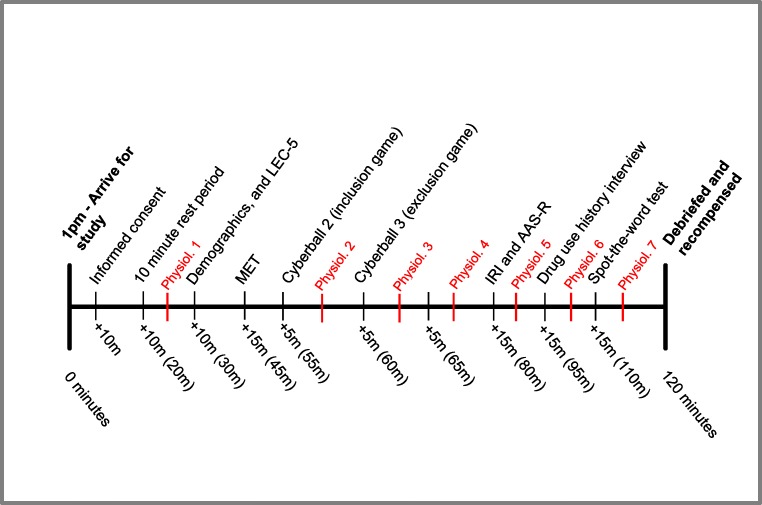
Study procedures in sequential order and accompanied by approximate timings. There were seven time points where physiological measures (salivary cortisol and blood pressure) were collected, and are labelled ‘Physiol.’ in red

Participants arrived in the afternoon between the times of 1 and 1.30 pm to control for diurnal variation in cortisol, and testing lasted for approximately 2 h. All procedures and approximate timings are depicted in Fig. 2.

Upon completion of all procedures, participants were fully debriefed on the true nature of the study, and given an opportunity to ask any questions. Participants were remunerated for their participation with a voucher.

### Statistical analysis

Data were analysed using the Statistical Package for Social Sciences (SPSS) version 23 and Mplus version 8. Assumptions of normality were checked, extreme outliers were winsorized (Wilcox 2005) and random missing values were imputed by group mean substitution.

A series of one-way, between-subject ANOVAs were used to assess the effect of group on both emotional and cognitive empathy. For the Cyberball Games, subjective responses to social exclusion were analysed using a series of 3 × 2 mixed measures ANOVAs assessed the effects of group and inclusion status on subjective measures (the PCQ and craving). For the cortisol and heart rate, latent growth curve models (LGCM) were used to understand the between-person difference in the trajectory of responses over time in respect to the average trend (Muthén and Curran 1997; Willett and Sayer 1994) and encompass features of both structural equation modelling and repeated measures ANOVA (Duncan et al. 2013) (described in SM2).

Any significant interactions were investigated further using post hoc *t* tests, which were adjusted using the Holm-Bonferroni correction. Differences between groups in demographic information was analysed using *t* tests, Chi-square tests where data was categorical and the Kruskal-Wallis test (groups > 3) or Mann-Whitney *U* (groups < 2) test where data was non-parametric. Pearson’s correlations were used to assess statistical relationships, and Spearman’s correlations were used when normality was violated.

#### Latent growth curve modelling (LGCM)

To investigate if the levels of opioid exposure (‘Group’) were associated with different physiological response trajectories throughout the tasks, we applied LGCM using Mplus (Muthén and Muthén 2000) (the growth model procedure is described in more detail in SM2). Model fit was assessed using the comparative fit index (CFI), the Tucker-Lewis index (TLI), the root mean squared error of approximation (RMSEA) and the standardised root mean square residual (SRMR). Improvements in the model were assessed using both the Bayesian information criterion (aBIC) and Akaike information criterion (AIC). The robust maximum likelihood estimation (MLR) was used for each model.

## Results

### Demographics and drug use (Table 1)

Groups were matched in age, gender, ethnicity, alcohol use, verbal IQ, baseline positive affect and familial history of substance abuse problems and mental health problems. There were differences in the number of diagnosed mental health problems, with increased incidence of mental health problems in the non-intoxicated opioid users compared to controls (χ^2^ = 11.13, *p* = .004), but no other differences (Holm-Bonferroni corrected). Although there was an overall group difference in age individuals left education and baseline negative affect, after correction for multiple comparisons, there were no group differences.

**Table 1:** Demographic information and drug use between groups (means and standard deviations)

		Intoxicated (*n* = 20)	Non-intoxicated (*n* = 20)	Controls (*n* = 24)	Test statistic	*P* value
Age		44.45 (11.51)	40.40 (10.04)	43.13 (12.83)	*F* = 0.64	.533
Gender (male, female, other)		12, 8, 0	14, 6, 0	13, 10, 1	χ^2^ = 2.56	.663
Ethnicity (Caucasian, Hispanic, mixed)		20, 0, 0	18, 1, 1	21, 0, 3	χ^2^ = 5.20	.267
Age left education		16.25 (1.55)	15.32 (3.79)	17.65 (3.25)	*F* = 3.26	.045*
Verbal IQ		47.35 (10.82)	44.89 (8.91)	48.83 (5.76)	*F* = 1.09	.342
Mental health problems (*n* = yes)		11	16	8	χ^2^ = 11.12	.004**
Diagnosis (*n*)	Depression	10	14	6		
	Anxiety	5	2	1		
	Other	0	2	1		
Physical health problems (*n* = yes)		6	4	3	χ^2^ = 2.04	.360
Antidepressants (*n* = yes)		7	10	5	χ^2^ = 4.72	.095
Oral contraceptives (*n* = yes)		1	1	0	χ^2^ = 1.71	.426
Familial mental health problems (*n* = yes)		4	6	9	χ^2^ = 1.61	.447
Familial substance use disorder (*n* = yes)		7	4	6	χ^2^ = 1.04	.595
Baseline positive affect		28.33 (7.50)	29.72 (8.17)	29.92 (7.13)	*F* = 0.25	.779
Baseline negative affect		14.16 (5.56)	15.45 (6.20)	11.71 (2.94)	*F* = 3.23	.046*
Opioid substitution medications (OSM)						
Medication, *n* (methadone, buprenorphine, other)		16, 1, 3	12, 6, 2		χ^2^ = 4.34	.114
Dose (standardised to oral morphine^b^, mg)		28.78 (17.24)	36.43 (19.32)		*F* = 1.75	.194
Months taken OSM		60.00 (173.25)^a^	12.00 (31.00)^a^		*U* = 106.0	.011*
Hours since taken OSM		3.92 (2.01)	23.41 (7.65)		*F* = 114.19	< .001***
Current regular drug use (*n*)						
Illicit opioids		9	9	0	χ^2^ = 15.03	.001
Alcohol		11	12	13	χ^2^ = 0.17	.919
Tobacco		14	17	7	χ^2^ = 15.46	< .001***
Cannabis		8	8	2	χ^2^ = 7.44	.024*
Benzodiazepines		3	3	0	χ^2^ = 3.97	.137
Cocaine		7	6	0	χ^2^ = 9.94	.007**
Salivary opioid screens		*n* = 20	*n* = 20			
Methadone, *n* = positive, % due to opioid prescription		16, 100%	13, 83.3%			
Buprenorphine, *n* = positive		0	1, 100%			
Opiates, *n* = positive		6, 33.3%	1, 0%			
Urine drug screens		*n* = 20	*n* = 15	*n* = 24		
Methadone, *n* = positive		14	10	0		
Opiates, *n* = positive		9	8	0		
Cannabis/THC, *n* = positive		6	5	3		
Cocaine, *n* = positive		5	5	2		
Amphetamine, *n* = positive		1	1	2		
Benzodiazepines, *n* = positive		3	7	0		
MDMA, *n* = positive		0	1	0		

Note: ^a^Non-parametric data: median and IQR are reported

^b^The equivalent doses are an approximation and calculated from the following sources (Foley 1985; Royal College of Anaesthetists 2018)

Current regular use of MDMA, amphetamines, and hallucinogens were excluded from the table due to minimal numbers

*p < .05, **p < .01, ***p < .001

There was a significant difference in the number of months taking an OSM between the two opioid user groups, with a greater number of months on OSM in the intoxicated (see Table 1). There were significant group differences in substance use for opioids, tobacco, cannabis and cocaine users comparing both the opioid groups to the controls (χ^2^ = 15.02, *p* = .012; χ^2^ = 14.53, *p* = .012; χ^2^ = 7.44, *p* = .042; and χ^2^ = 9.79, *p* = .016, respectively); however, there were no significant differences in illicit substance use between the two opioid user groups (χ^2^ = 0.00, *p >* .999*,* χ^2^ = 1.29, *p* > .999; χ^2^ = 0.00, *p* > .999; and χ^2^ = 0.11, *p* > .999, respectively) (Holm-Bonferroni corrected). Further details on drug use history can be found in SM3.

### Hypothesis 1: impairment in emotional empathy

#### The multifaceted empathy test (MET)

**Fig. 3:**
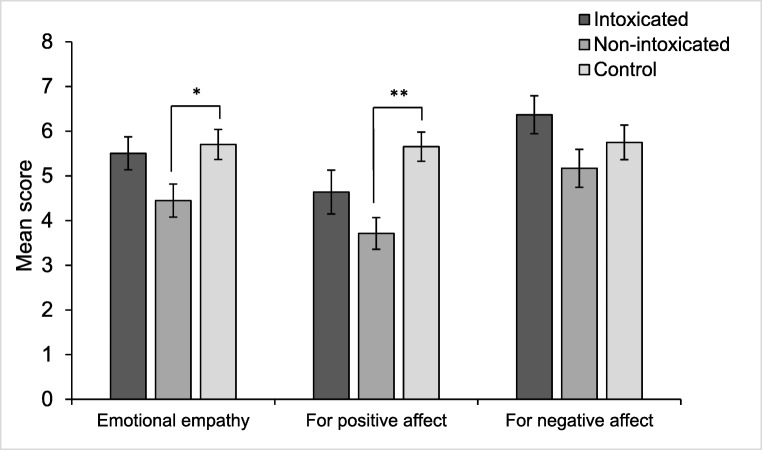
Emotional empathy on the MET between the three groups. There were significantly lower emotional empathy overall in the non-intoxicated opioid user group compared with the controls (*p < .05). When broken down into positive and negative affect, there were significant lower levels of emotional empathy for positive emotions in the non-intoxicated user group compared with controls (**p < .01); however, there were no differences between the intoxicated users and controls, or any group differences in negative affect

For emotional empathy, there was a significant difference in group (*F*(2,61) = 3.52, *p* = .036, *η*^2^ = .10). Holm-Bonferroni *t* tests indicated the non-intoxicated user group scored significantly lower than the controls (*t*(42) = 2.64, *p* = .048, η^2^ = .14) (Fig. 3); however, there were no differences between the non-intoxicated users and the intoxicated users (*t*(38) = 1.91, *p* = .128, η^2^ = .09) or the intoxicated users and controls (*t*(42) = 0.40, *p* = .688, η^2^ < .01). Emotional empathy to either positive or negative affect was also explored (all analyses were Holm-Bonferroni corrected). For positive affect, there was an effect of group (*F*(2,61) = 6.39, *p* = .024, η^2^ = .17), where the non-intoxicated group rated significantly lower than controls (*t*(42) = 4.03, *p* = .002, η^2^ = .28). There were no differences between the intoxicated and non-intoxicated users (*t*(38) = 1.53, *p* = .512, η^2^ = .06) or intoxicated users and controls (*t*(42) = 1.78, *p* = .415, η^2^ = .07) (Fig. 3). For negative affect, there were no differences between groups (*F*(2,61) = 1.99, *p* = .512, η^2^ = .06).

When assessing cognitive empathy, there were no significant differences between the three groups (*F*(2,56) = 1.76, *p* = .182, η^2^ = .04). Number of words known in the MET was included as a covariate in this analysis due to being correlated with cognitive empathy (*r* = .55, *n* = 60, *p* < .001). There were no group differences in cognitive empathy for positive or negative affect (*F*(2,61) = 1.07, *p* = .696, η^2^ = .03, and *F*(2,61) = 1.03, *p* = .696, η^2^ = .03, respectively) (Holm-Bonferroni corrected). For M and SDs for MET subscales, see SM4.

#### The Interpersonal Reactivity Index (IRI)

For emotional empathy subscales, there were no significant group differences in ‘empathic concern’ (*F*(2,61) = 0.14, *p* = .871, η^2^ = .01) or ‘personal distress’ (*F*(2,61) = 0.05, *p* = .950, η^2^ < .01). For cognitive empathy subscales, there were no significant group differences in ‘perspective taking’ (*F*(2,61) = 0.95, *p* = .394, η^2^ = .03) or ‘fantasy’ (*F*(2,61) = 1.62, *p* = .206, η^2^ = .05) (for M and SDs, see SM4).

### Hypothesis 2: chronic opioid users show dampened responses to social pain

#### The Cyberball Task

There were significant main effects of inclusion status which reflected decreases in mood, self-esteem, control, meaningful existence and sense of belonging following exclusion, as well as increases in hurt feelings. However, there were no significant effects of group, or interaction between inclusion status and group (Table 2).

**Table 2:** Statistical outcomes for the Cyberball Subscales and opioid craving

	Inclusion status	Intoxicated	Non-intoxicated	Control	F-statistic		*p* value	η^2^
**∆**Mood	Inclusion	2.49 (1.04)	2.27 (1.35)	2.62 (0.83)	Group	1.95	.151	.03
	Exclusion	1.65 (1.92)	0.36 (2.03)	0.95 (1.74)	Inclusion status	39.00	< .001***	.18
					Group*inclusion status	1.79	.176	.02
Self-esteem	Inclusion	3.02 (1.33)	2.80 (1.23)	3.44 (0.95)	Group	1.63	.205	.09
	Exclusion	2.41 (1.31)	1.87 (0.85)	2.25 (0.89)	Inclusion status	47.28	< .001***	.39
					Group*inclusion status	1.68	.196	.03
Sense of belonging^a^	Inclusion	1.35 (0.48)	1.32 (0.71)	1.14 (0.28)	Group	0.77	.466	.02
	Exclusion	2.45 (1.43)	2.92 (1.48)	2.53 (1.13)	Inclusion status	69.46	< .001***	.52
					Group*inclusion status	0.74	.480	.01
Meaningful existence^a^	Inclusion	0.09 (1.78)	0.11 (0.16)	0.03 (0.08)	Group	2.23	.116	.03
	Exclusion	0.34 (0.22)	0.35 (0.26)	0.26 (0.21)	Inclusion status	52.13	< .001***	.28
					Group*inclusion status	< .01	.996	< .01
Control^a^	Inclusion	0.30 (0.20)	0.31 (0.20)	0.38 (0.17)	Group	1.06	.352	.02
	Exclusion	0.18 (0.22)	0.09 (0.16)	0.16 (0.20)	Inclusion status	68.12	< .001***	.20
					Group*inclusion status	1.97	.148	.01
Anger	Inclusion	1.15 (0.67)	1.40 (0.88)	1.04 (0.20)	Group	12.12	< .001***	.24
	Exclusion	1.15 (0.37)	2.50 (1.32)	1.46 (0.78)	Inclusion status	14.22	< .001***	.13
					Group*inclusion status	5.42	.007	.10
Hurt feelings^a^	Inclusion	0.02 (0.07)	0.02 (0.11)	0.02 (0.08)	Group	0.20	.822	< .01
	Exclusion	0.19 (0.27)	0.23 (0.27)	0.19 (0.25)	Inclusion status	32.25	< .001***	.19
					Group*inclusion status	0.09	.910	< .01
% of perceived ball throws	Inclusion	32.93 (10.75)	38.62 (23.12)	41.47 (19.81)	Group	0.91	.409	.01
	Exclusion	15.27 (9.87)	11.14 (6.24)	16.03 (11.18)	Inclusion status	62.61	< .001***	.39
					Group*inclusion status	0.97	.386	.01
**∆**Mood^b^ (baseline to exclusion)	Baseline	14.35 (8.91)	13.83 (11.05)	18.21 (9.52)	Group	0.68	.508	.02
	Exclusion	7.22 (14.06)	6.44 (12.09)	8.58 (12.41)	Inclusion status	32.06	< .001***	.11
					Group*inclusion status	0.43	.652	< .01

Note: **∆**Mood was calculated by subtracting negative affect scores from overall positive affect scores. The adjectives used to compute positive mood in the PCQ were good, happy, friendly, relaxed, whilst negative mood were bad, sad, unfriendly and tense

^a^Log transformation was applied. Mean values are adjusted for the log transformation

^b^**∆**Mood (baseline to exclusion) is a manipulation check that Cyberball exclusion condition caused reductions in mood from baseline (using responses on the PANAS rather than mood assessed by the PCQ)

**Fig. 4:**
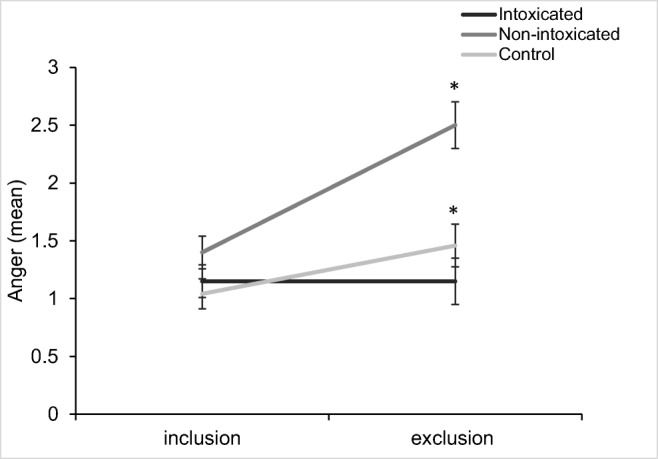
Anger following the inclusion and exclusion games between the three groups. Both the non-intoxicated opioid user group and the controls significantly increase in anger from inclusion to exclusion, whilst the intoxicated opioid user group remain the same. There was also a significant main effect of inclusion status, and a significant main effect of group (*p < .05)

For Anger, there was a significant interaction between inclusion status and group (*F*(2,61) = 5.42, *p* = .007, η^2^ = .10). Holm-Bonferroni corrected pairwise comparisons indicated that there was a significant difference in anger between the non-intoxicated user group with the intoxicated group (*p* < .001) and controls (*p* < .001); however, there were no differences between the intoxicated group with controls (*p* = .561) (Fig. 4). There was also a main effect of inclusion status (*F*(1,61) = 14.11, *p* < .001, η^2^ = .13), alongside a main effect of group (*F*(2,61) = 12.12, *p* < .001, η^2^ = .24). There were no effects of group or inclusion status on opioid craving (SM5).

### Physiological responses

#### Salivary cortisol

**Fig. 5:**
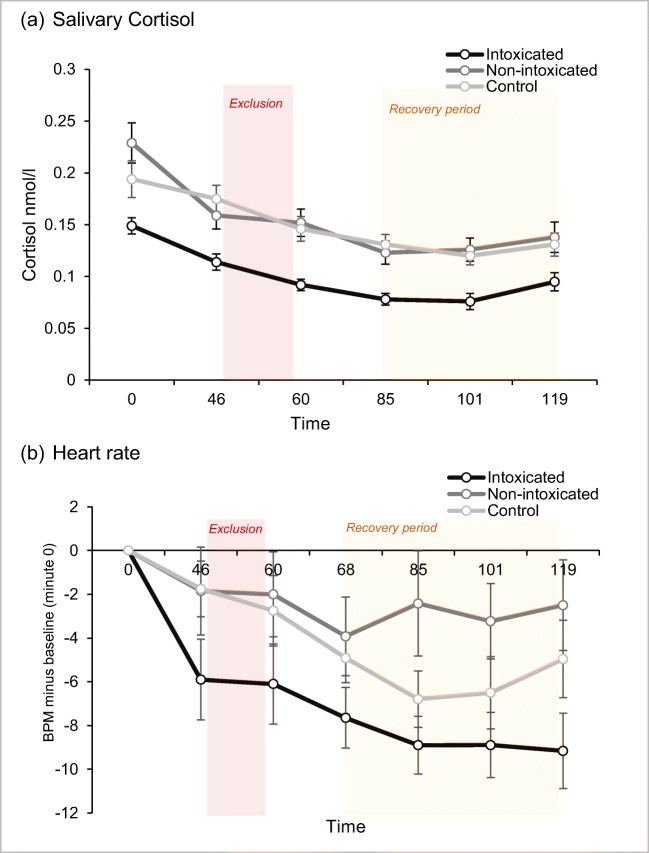
Physiological responses between the three groups over the seven time points

The LGCM with continuous latent variables of intercept for cortisol at minute 0 (baseline) and a quadratic slope as outcome between minutes 0 and 119 including dummy-coded group as the covariate revealed a good fit χ^2^(22) = 34.54, *p* = .043, CFI = .94; TLI = .93; SRMR = .07; RMSEA = .09, 90%CI [0.02,0.15]; AIC = − 1064.34; aBIC = − 1027.66. Being intoxicated was negatively related with the intercept at 0 minutes (*b* = − 0.07, SE = 0.03, *p* = .016), suggesting they had lower cortisol levels at baseline compared to the controls, but there were no effects for the non-intoxicated group (*b* = − 0.01, SE = 0.04, *p* = .759) who showed similar cortisol levels as the controls (Fig. 5a). In addition, there were significant effects of the intoxicated group when the intercept was set at minutes 46 (post-inclusion), 60 (post-exclusion), 85 (recovery period) and 101 (recovery period) (see SM6 for the data) indicating that intoxicated users had lower cortisol responses throughout social exclusion and recovery in comparison to the non-intoxicated and controls. Neither being intoxicated (*b* = 0.01, SE = 0.01, *p* = .326) nor being non-intoxicated (*b* < 0.01, SE = 0.01, *p* = .690) was associated with the slope, suggesting that the trajectory of cortisol over time was not associated to acute opioid state.

#### Heart rate

A piecewise LGCM with continuous latent variables of intercept, with one linear slope from 46 to 68 min (the Cyberball paradigm) and the second linear slope from 85 to 119 min (post-exclusion recovery period) in heart rate change, including group and interpersonal trauma as a covariate revealed the best and an overall acceptable fit χ^2^(21) = 36.73, *p* = .018, CFI = .95; TLI = .92; SRMR = .04; RMSEA = .12, 90%CI [0.05, 0.18]; AIC = 2008.64; aBIC = 2056.81. Being intoxicated had a significant negative effect on the intercept at 46 min (*b* = − 4.77, SE = 2.17, *p* = .028) (Fig. 5b), suggesting lower heart rate at baseline. In addition, there were significant effects of the intoxicated group when the intercept was set at minutes 60 and 101 and minutes 68 and 119 (see SM6) indicating that intoxicated users had less change in heart rate throughout social exclusion and recovery in comparison to the non-intoxicated and controls. There were no significant slope effects but the intoxicated user group had a near-significant effect on the linear slope between 46 and 68 min (*b* = 1.04, SE = 0.55, *p* = .057), and a similar trend was observed in the non-intoxicated user group (*b* = 1.26, SE = 0.71, *p* = .075) suggesting a gentler downward slope compared with the control condition. There was also a trend to suggest the effect of the intoxicated user group on the linear slope between 85 and 119 min (*b* = − 1.46, SE = 0.84, *p* = .081), suggesting smaller change during the recovery period compared with the controls. Rates of interpersonal trauma did not exert any significant effects on the intercept or slopes although adding it improved overall model fit.

### Exploratory analyses

Emotional empathy was not correlated with the total months taking an OSM (*r*^s^ = − .372, *n* = 20, *p* = .424) or hours since the OSM was taken (*r* = − .159, *n* = 15, *p* > .999) within the non-intoxicated group, nor was it correlated with rates of mental health problems over the sample (*r* = .03 *n* = 63, *p* < .999). There was a medium effect size for the association between emotional empathy and OSM dose within the non-intoxicated user group; however, it failed to reach significance (*r* = − .49, *n* = 20, *p* = .203).

Negative affect at baseline was not significantly related to emotional empathy for positive emotions (*r* = − 0.04, *n* = 63, *p* = .773) or change in anger from inclusion to exclusion (*r* = − 0.07, *n* = 63, *p* = .606).

## Discussion

The current study aimed to assess empathy and responses to social exclusion among individuals with opioid use disorder. We found lower emotional empathy (i.e., the ability to vicariously experience the emotional state of others) among non-intoxicated opioid users compared with opioid-naïve controls, particularly for positive emotions. Non-intoxicated opioid users also expressed significantly greater anger after being socially excluded compared to the intoxicated user group and controls. On the other hand, intoxicated opioid users showed lower salivary cortisol and heart rate across the testing session; however, they did not differ in the level at which cortisol and heart rate particularly increased or decreased in response to social exclusion.

The finding of lower emotional empathy in the non-intoxicated users partially replicates previous research suggesting impaired empathy among drug users (Ferrari et al. 2014; Kroll et al. 2018a, 2018b; Maurage et al. 2011; Preller et al. 2014) and opioid users specifically (Kroll et al. 2018a; Stange et al. 2017; Tomei et al. 2017), but crucially highlighted that acutely intoxicated opioid users show intact emotional empathy compared to controls. This was contrary to our initial prediction that empathy would be lowest within the intoxicated user group. Previous work in healthy participants has connected higher levels of endogenous opioids with decreased empathy for pain, possibly due to a decreased sensitivity in the ability to feel pain in oneself (Rutgen et al. 2015); therefore, it has been suggested that the use of analgesic drugs like opioids could also reduce empathy. Our results suggest that this is not the case in this group of chronic opioid users and, in fact, the on-board opioids appear to repair their empathy to the level of controls, whereas non-intoxicated users showed impairments—specifically for positive emotions. There was a medium to large effect size for the correlation between opioid substitution medication (OSM) dose and emotional empathy, potentially indicating a dose-dependent reduction in emotional empathy within the non-intoxicated group that was not driven by outliers. However, this should be interpreted with caution, as this relationship was using a small sample size and was non-significant after adjusting the α-criterion for multiple comparisons. Further work should investigate whether there is a dose dependent relationship between opioid use and empathy.

The specific impairment in empathy for positive emotions was demonstrated previously in a similar study with opioid users (Kroll et al. 2018a). This suggests a possible negative bias where relating to positive emotions is more difficult for opioid users not currently experiencing the acute effects of opioids. Prior research has suggested that abstinent opioid addicts are biased when attending to negative emotions, where they show enhanced detection of negative expressions during a visual search paradigm (Zhou et al. 2012). This bias is potentially due to greater exposure to negative expressions and reactions from society in everyday life, as well as impaired emotion processing that could predate addiction (Zhou et al. 2012). Additional to this, distress intolerance—the inability to endure difficult emotional states—is associated with greater attentional bias towards negative emotions and decreased attention toward positive emotions (Macatee et al. 2018). Opioid users on an OSM show greater distress intolerance (Kathryn McHugh and Otto 2012), where opioids may heighten the threshold to cope with difficult emotional states. The empathy deficit for positive emotions in the non-intoxicated users may therefore be due to reduced exposure to positive emotions in everyday life and reduced attention towards them. Opioid intoxication may serve to remediate emotion difficulties by increasing distress tolerance and enhancing their ability to relate to positive emotions.

The study also reported a novel finding of increased rates of anger following social exclusion in the non-intoxicated opioid users, compared to the intoxicated user group and controls. Past research has linked anger expression with endogenous opioid functioning, suggesting that increased anger expression may be related to an impaired endogenous opioid response to stress (Bruehl et al. 2007). Preclinical evidence supports this assumption, finding that opioid blockade using naltrexone has shown to increase rates of anger and pain (Bruehl et al. 2008; Burns et al. 2009). As the non-intoxicated user group in the current study may have a dampened endogenous opioid response, this could possibly account for the large increase in anger after being socially excluded. The intoxicated user group may experience no change in anger due to the acute effects of opioids buffering from this unpleasant emotional state. Higher rates of hostility and anger are related to poor emotion regulation in drug users (Handelsman et al. 2000; Shabanloo et al. 2018). This finding of greater anger, together with impaired empathy, potentially suggests an overall impairment in both understanding and expressing one’s own emotions in individuals who have chronically used opioids but are not acutely under the influence of them. It could suggest that opioids are used to alleviate difficult emotional states such as anger, and heighten users’ ability to tolerate social exclusion.

Cortisol and heart rate change were lower in the intoxicated user group, which is to be expected given cardiac depression following opioids (Vargish et al. 1987) and evidence that opioids can reduce cortisol responses to psychosocial stress (Bershad et al. 2015). Heart rate did not recover (reduce) over the duration of the experiment for the non-intoxicated user group; prior work has indicated the role of the endogenous opioid system in the recovery of the cardiovascular response to stress by reducing heart rate and cortisol (Morris et al. 1990). Heart rate has been linked with emotional and cognitive functions, where lower heart rate variability is related with poorer emotion regulation, higher alcohol craving (Ingjaldsson et al. 2003), and lower empathy (Lischke et al. 2018). Moreover, the groups did not differ in physiological responses to social exclusion and over the recovery period as we expected. A psychosocial stressor such as the Trier Social Stress Test (Kirschbaum et al. 1993) may produce more robust changes in cortisol and heartrate.

The current findings on empathy broadly concur with impairments observed in a previous study of opioid users by Kroll and colleagues (2018a) who also implemented the Multifaceted Empathy Test (MET); however they reported impairments in *cognitive* empathy (i.e. the ability to understand and identify the emotional states of others) among non-medically prescribed opioid users. The discrepancy between the two studies could be due to various differences between our samples: the sample tested by Kroll et al. excluded those with history of heroin abuse, and consequently may have experienced much lower levels of deprivation, poly-drug use and social adversity than our sample. One similarity between the two studies is specific impairment to positive emotions, which could suggest an overall negativity bias across the samples irrespective of socioeconomic or drug use background.

The study had limitations. Firstly, the intoxicated group had been prescribed OSM for more months than the non-intoxicated group; however, the months on OSM were not correlated with empathy, and the impairment in empathy was within the non-intoxicated group which suggests that this does not account for the key findings of the study. Secondly, the study did not measure symptoms of opioid withdrawal; however, this could have been linked with increased distress and anger following rejection in the non-intoxicated group. In addition, high rates of polysubstance and antidepressant use were reported in the opioid user groups, which could have biased the results. Nonetheless, the two opioid groups are well matched in drug use history which indicates a specific effect of opioid intoxication on emotional empathy and post-exclusion anger. The three groups were well matched in other variables, including loneliness and history of childhood adversity.

In summary, the current study provides both novel findings and supporting evidence for altered social functioning among opioid users. Blunted subjective anger in response to stress and lower cortisol and heart rate was observed in intoxicated users, which partially supports the notion that opioids could cause hyperalgesia to social pain. Impaired emotional empathy and increased rates of anger among opioid users who are not currently intoxicated with opioids may be due to an attentional bias toward negative expressions and poorer ability to tolerate difficult emotions, which is repaired by the use of opioids. With this knowledge, potential treatments for opioid use disorder should focus on heightening one’s ability to tolerate difficult social situations in a wider attempt to improve social skills, alongside emotion regulation training specifically aimed at reducing anger.

## Electronic supplementary material

ESM 1 (DOCX 16 kb)

ESM 2 (DOCX 12 kb)

ESM 3 (DOCX 16 kb)

ESM 4 (DOCX 13 kb)

ESM 5 (DOCX 15 kb)

ESM 6 (DOCX 23 kb)

ESM 7 (DOCX 15 kb)
