# Supplementary material for: Impaired empathy and increased anger following social exclusion in non-intoxicated opioid users
Source: Psychopharmacology (Berl). 2019 Nov 5;237(2):419–30. doi: 10.1007/s00213-019-05378-x (PMC7018792; doi:10.1007/s00213-019-05378-x)
Supplement: Supplementary file 1 — (DOCX 16 kb) [file 213_2019_5378_MOESM1_ESM.docx]

**Supplementary Material**

**SM1**

*Post-ostracism Cyberball Questionnaire (Williams et al., 2002)*. This 25-item scale assesses the following: Mood; belongingness; self-esteem; control; meaningful existence; anger; hurt feelings; % of perceived ball throws (manipulation check). Pre- and post-exclusion Responses were recorded on a 5-point Likert scale (1 = not at all; 5 = very much).

*Interpersonal Reactivity Index (Davis, 1980).* This 28-item scale assesses trait empathy, and differentiates between self-reported cognitive and emotional empathy. It consists of four different subscales, two of which characterising emotional empathy (empathic concern; personal distress), and the subsequent two characterising cognitive empathy (perspective taking; fantasy scale). Responses were recorded on a 5-point Likert scale (A = does not describe me well; E = describes me very well).

*Craving.* A 100mm visual analogue scale (VAS) was used to assess craving for opioid drugs following each Cyberball game. It included three, single items that assessed drug liking, wanting, and motivation to obtain opioid drugs – a method of which has been frequently used in the past with high validity (Pool, Sennwald, Delplanque, Brosch, & Sander, 2016). The term ‘opioid drugs’ was used to cover craving for any opioid-acting drug; such as methadone, buprenorphine, and heroin.

*The Life Events Checklist Version 5 (Weathers et al., 2013).* This 17-item questionnaire assesses whether participants have been previously exposed to any stressful or traumatic life events, and how proximal these events were to the participant (‘happened to me’; ‘witnessed it’; ‘learned about it’; ‘part of my job’; ‘not sure’; ‘doesn’t apply’). This questionnaire was adapted in the current study to include age when event occurred so that responses could be categorised into childhood, pre- to mid-adolescence, and adult trauma. Responses were further categorised into interpersonal (e.g. physical or sexual abuse) and non-interpersonal (e.g. transportation accident, or fire).

*UCLA Loneliness Scale (Russell, 1996).* This 20-item scale assesses feelings of social isolation and loneliness. Responses were recorded on a 4-point scale (1 = never; 4 = often).

*Spot-the-Word Test (Baddeley, Emslie, & Nimmo-Smith, 1993).* This test was used to assess verbal IQ by presenting participants with 60 word-pairs. In each pair, one word was real and one word was made up, and participants were asked to identify the real word in each pair.

*Positive and Negative Affect Schedule (Watson, Clark, & Tellegen, 1988)*. This 20-item questionnaire differentiates between positive and negative mood states using a 5-point scale (1 = very slightly or not at all; 5 = extremely). Positive and negative affect are computed by adding the scores for either subscale, and was used to assess mood at baseline.

*Physiological Measures*. Seven saliva samples were collected by passive drool method using Cryovial 3.5mL collection tubes. Participants were required to provide approximately 2ml of saliva, which was immediately stored at -80°C until analysis. Saliva samples were analysed using enzyme-linked immunosorbent assay (ELISA) kits to assess cortisol levels, (Salimetrics) with an assay sensitivity of <0.007 ug/dL, as well as levels of methadone, buprenorphine, and opiates (Immunalysis) with a sensitivity of 5ng/mL, 1ng/mL, and 10ng/mL, respectively. All samples were analysed in duplicate. Other measures of physiological arousal included heart rate, which was assessed each time a saliva sample was taken using an automatic blood pressure monitor (Omron M3 IT Intellisense), where a cuff was placed around the upper arm of the participant.
