# Supplementary material for: Impaired empathy and increased anger following social exclusion in non-intoxicated opioid users
Source: Psychopharmacology (Berl). 2019 Nov 5;237(2):419–30. doi: 10.1007/s00213-019-05378-x (PMC7018792; doi:10.1007/s00213-019-05378-x)
Supplement: Supplementary file 2 — (DOCX 12 kb) [file 213_2019_5378_MOESM2_ESM.docx]

**SM2**

*Statistical Analysis: Latent Growth Curve Modelling.* We fitted growth models in which repeated measures of either heart rate (controlled for baseline) or cortisol represent indicators of continuous latent variables, growth factors, the intercept (i.e., mean starting value) and the linear (i.e., rate of growth) and quadratic (i.e., levelling off, or coming down) slopes. In order to understand the role of opioid exposure, we added dummy-coded variables ‘intoxicated’, ‘non-intoxicated’ and ‘controls’ as covariates to our growth-curve model. Resulting coefficients signify the contribution of each respective opioid level in the context of all other opioid level groups. Interpersonal trauma was added as an additional covariate for heart rate due to improving overall model fit, despite not having an independent contribution. We centred the intercept at minute 0 (baseline) for cortisol and minute 46 for heart rate (baseline-corrected), but also ran alternative models with differing centre points from minute 46-119 for cortisol and minute 60-119 for heart rate to describe the influence of opioid exposure at different times during the exercises (54).

Time point four (minute 68) was excluded from the model for cortisol due to severe interferences with model fit, causing non-convergence (implications are discussed).

In addition, when analysed using ANOVA’s, there were no Group differences in AUCg and AUCi (F(2,61)=0.20, *p*=.823, η²=.01 and F(2,61)=0.30, *p*=.740, η²=.01, respectively.
