# Supplementary material for: Impaired empathy and increased anger following social exclusion in non-intoxicated opioid users
Source: Psychopharmacology (Berl). 2019 Nov 5;237(2):419–30. doi: 10.1007/s00213-019-05378-x (PMC7018792; doi:10.1007/s00213-019-05378-x)
Supplement: Supplementary file 3 — (DOCX 16 kb) [file 213_2019_5378_MOESM3_ESM.docx]

**SM3**

Detailed drug use history between the three groups. The data for each substance reported in the following table is only for the individuals who expressed using that drug regularly (either in the past and/or currently). The reported data includes: the total number of years the substance was used for, number of days the substance was used per month, and the amount of substance used per session.

|  | **Intoxicated (n=20)** | **Non-intoxicated (n=20)** | **Controls (n=24)** |
| --- | --- | --- | --- |
| **Illicit opioids (n=ever used regularly)** | **18** | **17** | **0** |
| Years used | 16.42 (11.29) | 12.03 (2.32) |  |
| Days used per month (before OSM) | 28.00 (0.00)^a^ | 28.00 (0.00)^a^ |  |
| Days used per month (after OSM) | 3.80 (7.73) | 5.64 (9.03) |  |
| Amount used per day (heroin, grams) | 0.30 (0.70)^a^ | 0.30 (0.30)^a^ |  |
| **Alcohol** | **16** | **14** | **16** |
| Years used | 23.31 (13.17) | 15.17 (9.51) | 22.42 (14.47) |
| Days used per month | 15.00 (20.00)^a^ | 28.00 (14.50)^a^ | 6.00 (12.00)^a^ |
| Amount used per session (units) | 13.50 (31.75)^a^ | 15.00 (19.00)^a^ | 12.00 (14.25)^a^ |
| **Tobacco** | **17** | **14** | **9** |
| Years used | 29.32 (10.42) | 20.08 (9.73) | 23.28 (14.11) |
| Days used per month | 28.00 (0.00)^a^ | 28.00 (0.00)^a^ | 28.00 (11.00)^a^ |
| Amount used per day (cigarettes) | 15.77 (7.96) | 15.72 (8.67) | 12.88 (9.57) |
| **MDMA** | **13** | **13** | **1** |
| Years used | 5.69 (3.90) | 4.79 (3.09) | 12.00 (n/a) |
| Days used per month | 7.79 (5.61) | 9.85 (6.72) | 4.00 (n/a) |
| Amount used per session (grams) | 2.98 (2.56) | 3.65 (2.77) | 0.50 (n/a) |
| **Cannabis** | **15** | **12** | **8** |
| Years used | 25.03 (13.53) | 19.08 (11.33) | 15.19 (11.56) |
| Days used per month | 28.00 (15.00)^a^ | 28.00 (0.00)^a^ | 16.50 (21.50)^a^ |
| Amount used per day (grams) | 1.80 (0.95) | 1.18 (0.59) | 1.05 (0.58) |
| **Amphetamines** | **14** | **8** | **5** |
| Years used | 5.50 (10.13)^a^ | 6.00 (11.00)^a^ | 7.00 (16.50)^a^ |
| Days used per month | 20.00 (21.00)^a^ | 28.00 (21.75)^a^ | 10.00 (15.50)^a^ |
| Amount used per session (grams) | 1.00 (1.00)^a^ | 3.50 (3.50)^a^ | 1.00 (0.56)^a^ |
| **Benzodiazepines** | **7** | **6** | **1** |
| Years used | 9.25 (20.00)^a^ | 2.00 (4.00)^a^ | 0.20 (n/a)^a^ |
| Days used per month | 5.00 (20.50)^a^ | 28.00 (12.13)^a^ | 28.00 (n/a)^a^ |
| Amount used per session (milligrams) | 15.00 (61.50)^a^ | 55.00 (295.25)^a^ | 80.00 (n/a)^a^ |
| **Cocaine** | **12** | **12** | **2** |
| Years used | 6.00 (21.25)^a^ | 3.75 (2.88)^a^ | 12.50 (n/a)^a^ |
| Days used per month | 16.00 (22.50)^a^ | 24.00 (23.00)^a^ | 15.00 (n/a)^a^ |
| Amount used per session (grams) | 0.83 (0.73)^a^ | 0.80 (1.75)^a^ | 1.38 (n/a)^a^ |
| *Note.* ^a^ non-parametric data: median and IQR are reported | | | |
