# Supplementary material for: Impaired empathy and increased anger following social exclusion in non-intoxicated opioid users
Source: Psychopharmacology (Berl). 2019 Nov 5;237(2):419–30. doi: 10.1007/s00213-019-05378-x (PMC7018792; doi:10.1007/s00213-019-05378-x)
Supplement: Supplementary file 4 — (DOCX 13 kb) [file 213_2019_5378_MOESM4_ESM.docx]

**SM4**

*Means and Standard Deviations for the Empathy Measures.*

|  | | | **Intoxicated** | **Non-intoxicated** | **Controls** |
| --- | --- | --- | --- | --- | --- |
| **Multifaceted Empathy Test (MET)** | | | | | |
| EE | | Total | 5.50 (1.80) | 4.45 (1.70) | 5.70 (1.46) |
|  |  | Positive affect | 4.64 (2.19) | 3.71 (1.59) | 5.65 (1.60) |
|  |  | Negative affect | 6.37 (1.83) | 5.17 (2.15) | 5.75 (1.71 |
| CE | | Total | 26.50 (3.19) | 24.60 (4.48) | 24.83 (3.56) |
|  |  | Positive affect | 15.85 (1.73) | 14.80 (2.78) | 14.96 (2.69) |
|  |  | Negative affect | 10.65 (2.25) | 9.80 (2.61) | 9.88 (1.36) |
| **Interpersonal Reactivity Index (IRI)** | | | | | |
| EE | Empathic concern | | 4.02 (0.68) | 3.91 (0.73) | 3.97 (0.68) |
|  | Personal distress | | 2.66 (0.80) | 2.61 (0.67) | 2.58 (0.76) |
| CE | Perspective taking | | 3.67 (0.71) | 3.36 (0.81) | 3.44 (0.71) |
|  | Fantasy | | 3.28 (0.95) | 2.81 (0.62) | 3.16 (0.99) |
| *Note*. EE denotes ‘emotional empathy’ and CE denotes ‘cognitive empathy’ | | | | | |
