# Supplementary material for: Impaired empathy and increased anger following social exclusion in non-intoxicated opioid users
Source: Psychopharmacology (Berl). 2019 Nov 5;237(2):419–30. doi: 10.1007/s00213-019-05378-x (PMC7018792; doi:10.1007/s00213-019-05378-x)
Supplement: Supplementary file 5 — (DOCX 15 kb) [file 213_2019_5378_MOESM5_ESM.docx]

**SM5**

Results for repeated measures ANOVAS on the effect of social exclusion on craving indices between the two opioid users, including ratings of 1) liking of opioids, 2) wanting opioids, and 3) motivation to use opioids:

|  | **Inclusion status** | **Intoxicated** | **Non-intoxicated** | **F-Statistic** | | ***p*** | **η²** |
| --- | --- | --- | --- | --- | --- | --- | --- |
| Opioid liking | Inclusion | 19.19 (26.48) | 17.00 (25.91) | Group | 0.05 | .827 | .03 |
|  | Exclusion | 20.42 (33.02) | 16.30 (26.24) | Inclusion status | 0.80 | .376 | .02 |
|  |  |  |  | Group*  inclusion status | 0.22 | .646 | .01 |
| Opioid wanting | Inclusion | 28.81 (35.04) | 20.15 (21.83) | Group | 0.07 | .797 | .03 |
|  | Exclusion | 29.94 (37.83) | 21.68 (23.55) | Inclusion status | 0.01 | .940 | <.01 |
|  |  |  |  | Group*  inclusion status | 0.31 | .580 | .01 |
| Opioid motivation | Inclusion | 3.87 (5.91) | 7.90 (22.09) | Group | 3.70 | .062 | .59 |
|  | Exclusion | 10.45 (16.37) | 12.18 (18.58) | Inclusion status | 1.10 | .302 | .01 |
|  |  |  |  | Group*  inclusion status | 0.10 | .758 | <.01 |
| *Note.* All analyses were log transformed due to deviations from normality. Means and standard deviations presented are the raw data. | | | | | | | |
