# Supplementary material for: Impaired empathy and increased anger following social exclusion in non-intoxicated opioid users
Source: Psychopharmacology (Berl). 2019 Nov 5;237(2):419–30. doi: 10.1007/s00213-019-05378-x (PMC7018792; doi:10.1007/s00213-019-05378-x)
Supplement: Supplementary file 6 — (DOCX 23 kb) [file 213_2019_5378_MOESM6_ESM.docx]

**SM6**

LGCM data for when the intercept is adjusted to multiple other time points in the model for both salivary cortisol and heart rate. Model one includes dummy coded groups ‘intoxicated’ and ‘non-intoxicated’ users, where the 'control’ group are excluded as a reference category. Model two includes dummy coded groups ‘intoxicated’ users and ‘controls’, where the non-intoxicated group is excluded as a reference category. For Model 2, only the beta, standard error and significance values for the ‘Intoxicated’ group are presented, as the values for controls are identical to the non-intoxicated user group in Model 1 (except that the direction of beta values are reversed). A summary for the model outcomes when the intercept is adjusted to each time point can be found below (i = intercept and s = slope).

| **Cortisol** | |  | | **Min 0** | | **Min. 46** | | | | **Min. 60** | | **Min. 85** | | **Min. 101** | | | **Min.119** | |
| --- | --- | --- | --- | --- | --- | --- | --- | --- | --- | --- | --- | --- | --- | --- | --- | --- | --- | --- |
|  |  |  |  | **i** | **s** | **i** | | **s** | | **i** | **s** | **i** | **s** | **i** | | **s** | **i** | **s** |
| **Model 1** | **Intoxicated** | ***b*** | | -.065 | .005 | -0.57 | | -.002 | | -.057 | .002 | -.053 | .006 | -.045 | | .010 | -.034 | .014 |
|  |  | **SE** | | .027 | .005 | .024 | | .011 | | .020 | .006 | .019 | .004 | .018 | | .009 | .023 | .014 |
|  |  | ***p*** | | .**016*** | .326 | **.016*** | | .864 | | **.005**** | .740 | **.004**** | .189 | **.012*** | | .264 | .138 | .340 |
|  | **Non-intoxicated** | ***b*** | | -.011 | .003 | -.003 | | -.008 | | -.009 | .002 | -.007 | .004 | <.001 | | .011 | .014 | .017 |
|  |  | **SE** | | .035 | .007 | .031 | | .014 | | .027 | .008 | .026 | .006 | .026 | | .011 | .031 | .017 |
|  |  | ***p*** | | .759 | .690 | .913 | | .541 | | .756 | .800 | .779 | .499 | .998 | | .324 | .653 | .320 |
| **Model 2** | **Intoxicated** | ***b*** | | -.054 | .002 | -.054 | | .006 | | -.049 | .004 | -.046 | .002 | -.045 | | .001 | -.048 | .003 |
|  |  | **SE** | | .028 | .006 | .026 | | .011 | | .023 | .006 | .022 | .006 | .024 | | .010 | .032 | .015 |
|  |  | ***p*** | | .054 | .761 | **.035*** | | .546 | | **.031*** | .532 | **.039*** | .795 | .061 | | .931 | .132 | .931 |
| **Heart rate** | | | **Min.’s 46 & 85** | | | | | | **Min.’s 60 & 101** | | | | | | **Min.’s 68 & 119** | | | |
|  |  |  | **i** | | **s1** | | **s2** | | **i** | | **s1** | | **s2** | | **i** | | **s1** | **s2** |
| **Model 1** | **Intoxicated** | ***b*** | 4.77 | | 1.04 | | -1.46 | | -5.18 | | 1.04 | | -1.46 | | -5.60 | | 1.04 | -1.46 |
|  |  | **SE** | 2.17 | | 0.55 | | 0.84 | | 2.30 | | 0.55 | | .836 | | 2.64 | | 0.55 | 0.84 |
|  |  | ***p*** | **.028*** | | .057 | | .081 | | **.024*** | | .057 | | .081 | | **.034*** | | .057 | .081 |
|  | **Non-intoxicated** | ***b*** | -0.48 | | 1.26 | | -0.48 | | 0.30 | | 1.26 | | -0.48 | | 1.08 | | 1.26 | -0.48 |
|  |  | **SE** | 2.14 | | 0.71 | | 0.97 | | 2.19 | | 0.71 | | 0.97 | | 2.60 | | 0.71 | 0.97 |
|  |  | ***p*** | .882 | | .075 | | .620 | | .892 | | .075 | | .620 | | .679 | | .075 | .620 |
| **Model 2** | **Intoxicated** | ***b*** | -4.28 | | -0.22 | | -0.98 | | -5.48 | | -0.22 | | -0.98 | | -0.36 | | -0.08 | -0.19 |
|  |  | **SE** | 2.65 | | 0.78 | | 1.04 | | 2.62 | | 0.78 | | 1.04 | | 0.15 | | .282 | 0.19 |
|  |  | ***p*** | .106 | | .783 | | .344 | | **.037*** | | .783 | | .344 | | **.013*** | | .786 | .299 |
| *Note.* Model 1 includes the dummy-coded variables ‘Intoxicated’ and ‘Non-intoxicated’ user groups, excluding ‘Controls’ as the reference category. Model 2 includes the dummy-coded variables ‘Intoxicated’ and ‘Controls’, excluding ‘Non-intoxicated’ users as the reference category.  Minute 0 = baseline, minute 46 = post-inclusion, minute 60 = post-exclusion, minute 68 = post-empathy (not analysed in this report), minute 85 = first recovery period, minute 101 = second recovery period, minute 119 = third recovery period.  **p*<.05 | | | | | | | | | | | | | | | | | | |
