# Supplementary material for: Impaired empathy and increased anger following social exclusion in non-intoxicated opioid users
Source: Psychopharmacology (Berl). 2019 Nov 5;237(2):419–30. doi: 10.1007/s00213-019-05378-x (PMC7018792; doi:10.1007/s00213-019-05378-x)
Supplement: Supplementary file 7 — (DOCX 15 kb) [file 213_2019_5378_MOESM7_ESM.docx]

**SM7**

When assessing trauma history, there was a trend to suggest a Group difference in interpersonal trauma during early childhood and adulthood, however this did not reach the statistical threshold for significance. There was an effect that approached significance to suggest a group difference in loneliness, with the intoxicated user group scoring the highest, followed by the non-intoxicated users and controls. There were no group differences in rates of non-interpersonal trauma.

|  | **Intoxicated users** | | **Non-intoxicated** | | **Controls** | **Test statistic** | ***p*-value** |
| --- | --- | --- | --- | --- | --- | --- | --- |
| Interpersonal trauma | | | | | | | |
| Childhood (ages 0-9), n=yes | 3 | | 6 | | 1 | χ²=5.53 | .063 |
| Pre-mid adolescence  (10-17) n=yes | 8 | | 9 | | 8 | χ²=0.64 | .728 |
| Adulthood (18+)  n=yes | 12 | | 14 | | 9 | χ²=4.98 | .083 |
| Non-interpersonal trauma | | | | | | | |
| Childhood  (ages 0-9) n=yes | 1 | 5 | | 5 | | χ²=3.17 | .205 |
| Pre-mid adolescence  (10-17) n=yes | 9 | 6 | | 5 | | χ²=2.99 | .225 |
| Adulthood (18+) n=yes | 11 | 15 | | 13 | | χ²=2.42 | .298 |
| Loneliness score | 53.33 (10.31) | 48.16 (9.36) | | 46.18 (10.35) | | F=2.78 | .070 |
| *Note*. Interpersonal trauma consisted of: Physical assault; sexual assault; unwanted sexual experiences; held captive; caused harm to others.  Non-interpersonal trauma consisted of: natural disasters; fire; transportation accident; serious accident; exposure to toxic chemicals; in combat; serious illness; observing human suffering; violent death; accidental death.  Age groups were based on: Wolitzky-Taylor, K., Sewart, A., Vrshek-Schallhorn, S., Zinbarg, R., Mineka, S., Hammen, C., ... & Craske, M. G. (2017). The effects of childhood and adolescent adversity on substance use disorders and poor health in early adulthood. *Journal of Youth and Adolescence, 46*(1), 15-27. | | | | | | | |
